# Preferred teaching styles of medical faculty: an international multi-center study

**DOI:** 10.1186/s12909-020-02358-0

**Published:** 2020-11-30

**Authors:** Nihar Ranjan Dash, Salman Yousuf Guraya, Mohammad Tahseen Al Bataineh, Mohamed Elhassan Abdalla, Muhamad Saiful Bahri Yusoff, Mona Faisal Al-Qahtani, Walther N. K. A. van Mook, Muhammad Saeed Shafi, Hamdi Hameed Almaramhy, Wail Nuri Osman Mukhtar

**Affiliations:** 1 Clinical Sciences Department, College of Medicine, University of Sharjah, Post Box –, 27272 Sharjah, United Arab Emirates; 2 Department of Medical Education, School of Medical Sciences, Universiti Sains Malaysia, Penang, Malaysia; 3 College of Public Health, Imam Abdulrahman Bin Faisal University, Dammam, Kingdom of Saudi Arabia; 4 School of Health Professions Education, Faculty of Health Medicine and Life Sciences, Maastricht University, Maastricht, the Netherlands; 5 Shifa College of Medicine, Shifa Tameer-e-Millat University, Islamabad, Pakistan; 6 College of Medicine, Taibah University, Almadinah Almunawwarah, Kingdom of Saudi Arabia; 7 Faculty of Medicine, University of Gezira, Gezira, Sudan

**Keywords:** Teaching style, Students learning style, Medical curriculum, Problem based learning, Instructional strategies.

## Abstract

**Background:**

In the current wave of educational reforms, understanding teaching styles of medical faculty can help modify instructional strategies for effective teaching. Few studies have probed distinctive teaching styles of medical faculty. We compared preferred teaching styles of faculty from seven medical schools in United Arab Emirates, the Netherlands, Saudi Arabia, Malaysia, Pakistan, and Sudan.

**Methods:**

The validated Grasha-Riechmann teaching style inventory was administered online for data collection and used SPSS version 20.0 for statistical analysis.

**Results:**

Of the 460 invitees, 248 responded (response rate; 54%). Delegator teaching style was most common with a highest median and mean of 2.38 and 2.45, respectively. There was a significant correlation between expert and authority teaching styles, correlation coefficient 0.62. Similarly, we found a significant correlation between authority teaching style and nature of curriculum, correlation coefficient 0.30. Multiple regression analysis showed that only authority teaching style and male gender had significant correlation. Interestingly, 117 (47%) teachers disagreed with the teaching philosophy of *delivering course contents by strictly following learning outcomes*. Female teachers (114/248) were more *willing to negotiate with their students regarding how and what to teach in their course*, while male teachers tended *to allow more autonomy by allowing students to set their learning agenda*.

**Conclusions:**

This study showed that the medical teachers preferred delegator teacher style that promotes students’ collaboration and peer-to-peer learning. Most teachers are conscious of their teaching styles to motivate students for scientific curiosity. These findings can help medical educators to modify their teaching styles for effective learning.

**Supplementary Information:**

The online version contains supplementary material available at 10.1186/s12909-020-02358-0.

## Background

Teaching styles also called teaching methods are principles, strategies and behaviors adapted by teachers to enable students’ learning. From a different perspective, teaching styles are reflected in how educators present themselves to learners, transfer learning material, interact with students, manage learning tasks, guide work in process, and engage students in their courses [1]. Teaching styles include an understanding about curriculum, students’ learning styles, academic performance, and professional knowledge [1, 2].

There is a great need to align teaching styles of medical faculty with the changing landscape of medical education due to ever increasing emphasis on integrated, problem-based, student-directed and peer-assisted horizontal collaborative learning methods [3]. Such changes have transformed the traditional authoritative role of teacher to more supervisory and mentoring conventions [4]. This has certainly created unease among traditional medical teachers who are entrenched with didactic lecturing, where they act as sole information providers with little interaction with the students [5]. The value of research in identifying individual teaching style to improve quality in academics is well documented [6]. The teachers who are conscious of their preferred teaching style would be able to recognize a diversity of teaching strategies as needed for different contexts and different students [2]. Similarly, a conscious recognition of individuals’ teaching styles fosters his knowledge and skills of teaching strategies, methods, appropriate use of technologies to organize learning-teaching processes more efficiently [7]. Most educators possess a unique teaching style that has a direct impact on educational environment [8]. Even within a single teaching-learning session, teachers may use a wide variety of teaching styles that helps align with students’ learning styles for successful learning outcomes [9, 10].

Several models of teaching styles inventories have been described in the literature. Among those, the inventory by Leung et al. contains four different teaching behaviours; assertive, suggestive, collaborative and facilitative [11]. Though this model showed high internal consistency and long-term test-retest reliability, so far its psychometric testing and predictive validity has not been analysed. From a different standpoint, Zhang has developed a 32-item Effective Teacher Inventory that provides self-reported analysis of teacher’s academic conceptions for effective teaching [12]. However, this inventory does not provide information about teachers’ behavioral or qualitative indicators of teaching styles. One popular model has been credited to the late Anthony F. Grasha, a psychology professor [13]. Grasha’s model includes five classic teaching styles: i. Expert: teacher is knowledgeable and subject expert by giving correct information to students, ii. Formal authority: teacher plays a role of manager who emphasizes acceptable and strict rules in guiding students, iii. Demonstrator: teacher behaves as a role model and encourages students to use one approach that is presumably effective in the teacher’s opinion, iv. Facilitator: teacher guides and directs students by asking questions, exploring options, suggesting alternatives, and encourages them to develop criteria to make informed choices, v. Delegator: teacher is concerned with students’ autonomy, expects learners to work independently and help them only on request. In the delegator style, individual teaching and learning styles are integrated that help to determine how teaching qualities of instructors and learners can improve learning experience [14].

Although several studies have utilized Garsha’s Teaching Style Inventory for determining teaching styles of their medical faculty [15–17] [18], no research has compared teaching styles of medical teachers from different medical schools. Comparison of teaching styles across several insitutions using similar curricula would provide valuable data for developing a standard teaching styles protocol for effective teaching. Understanding teaching styles of medical teachers will not only augment pedagogical competencies, but would also help teachers to stay current with their teaching strategies. We conducted this study to explore and compare teaching styles of medical academics from seven medical schools using problem-based learning (PBL) curriculum from the United Arab Emirates, the Netherlands Saudi Arabia, Malaysia, Pakistan, Sudan and. We expect that such data will help medical educators to align their instructional pedagogies in harmony with teaching styles.

## Methods

### Setting

We conducted this cross-sectional study after obtaining necessary institutional ethics approval (REC-18-04-15-01). We invited seven medical schools from six different countries to participate in this study. The participating universities were University of Sharjah (UoS) United Arab Emirates, Universiti Sains Malaysia (USM) Malaysia, Imam Abdulrahman Bin Faisal University (IAU) Saudi Arabia, Maastricht University (MU), The Netherlands, Shifa Tameer-e-Millat University (STMU) Pakistan, Taibah University (TU) Saudi Arabia and University of Gezira (UoG) Sudan. We selected these universities based on the commonalities in their undergraduate medical teaching. All these participating institutions practice an integrated PBL curriculum with similar instructional methods such as small group teaching, e-learning, clinical skills training, community-oriented education, and early clinical exposure. On average, medical program spans over 6 years and all invited faculty from the seven medical schools are actively involved in teaching. The invited faculty represented all departments of basic and clinical sciences and family and community medicine, and were currently teaching students of years one to six of their medical schools. We excluded the teachers working primarily in research labs without teaching responsibility in this research.

### Design

Teaching styles of the faculty in participating institutions were captured using the validated teaching style inventory developed by Grasha-Riechmann [6].

The inventory was administered online using Google® Inc. software. The Grasha-Riechmann teaching styles inventory contains 40 close-ended statements arranged in five categories: expert, formal authority, demonstrator, facilitator and delegator. In turn, each category contains eight different items as shown in **Supplementary Table** **1**.

### Statistical analysis

We performed descriptive analysis by summary statistics, which showed mean, median, standard deviation and maximum values of teaching styles, demographic and academic variables. We also perfoemed the pearson corrrelation analysis of teaching styles, demographic and academic variables. Finally, we performed multiple regression analysis for determining the relationships of each teaching style with demographic and academic variables. *The p-*values less than or equal to 0.01 and 0.05 were considered statistically significant. We used SPSS version 20.0 for all statistical analyses.

## Results

**Fig. 1:**
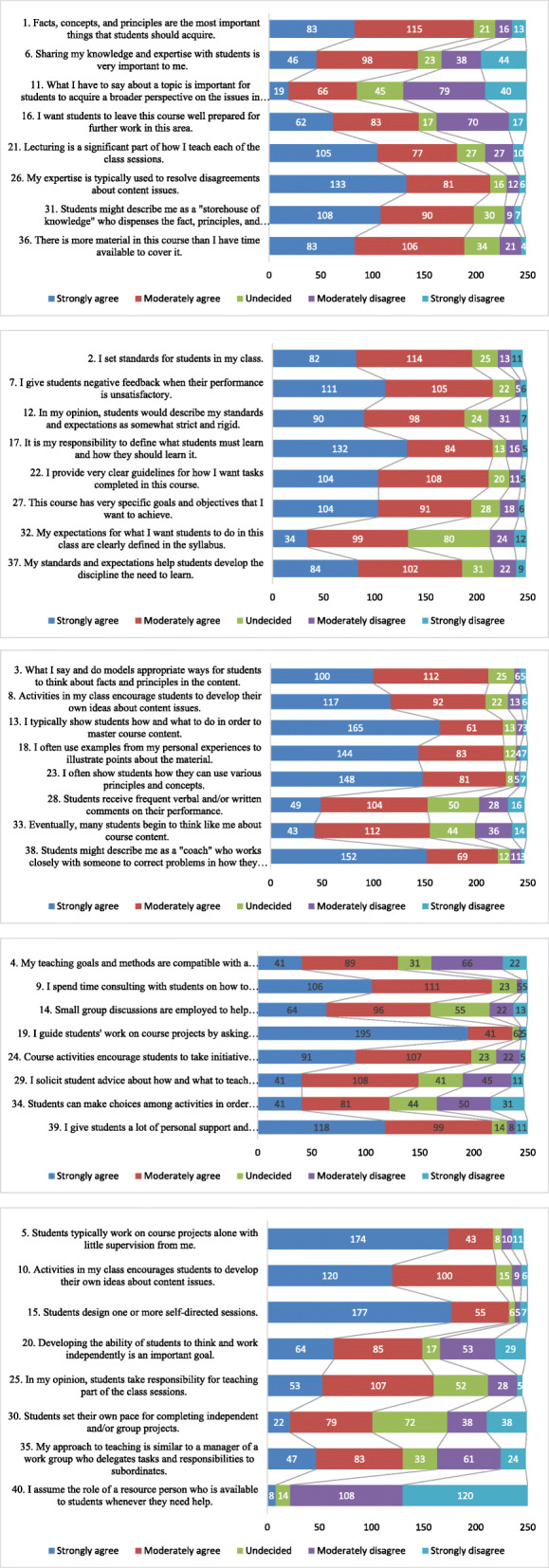
**a**: Clustered bar chart of observed frequencies of responses to statements about expert teaching styles (*N* = 248). **b**: Clustered bar chart of observed frequencies of responses to statements about authority teaching styles (*N* = 248). c: Clustered bar chart of observed frequencies of responses to statements about demonstrator teaching styles (*N* = 248). d: Clustered bar chart of observed frequencies of responses to statements about facilitator teaching styles (*N* = 248). e: Clustered bar chart of observed frequencies of responses to statements about delegator teaching styles (*N* = 248)

Of the 460 invitees, we received 248/460 complete responses (*N* = 248, response rate of 54%): 67 from IAU, 48 MU, 48 UoG, 34 TU, 24 UoS, 16 USM and 11 teachers from STMU. In terms of academic ranks, the cohort included 96 assistant professors, 53 associate professors, 39 full professors, 33 instructors, 13 lecturers, 8 non-faculty members, and 6 adjunct faculty members. There were 134 male and 114 female teachers; 136 had more than 10 years and 112 had less than 10 years teaching experience. Sixty-nine faculty members were assigned for teaching 5th year students, 33 for 4th year, 68 for 3rd year, 52 for 2nd year, and 26 were involved in teaching 1st year students. Fig. 1**a-e** show clustered bar chart of the observed frequencies in responses to different teaching styles (e.g., strongly agree, moderately agree, undecided, moderately disagree, and strongly disagree). Regarding *S19, I guide students’ work on course projects by asking questions, exploring options, and suggesting alternative ways to do things.’* we observed that the majority 195 (68.66%) strongly agreed to facilitate their students’ learning by allowing them to ask questions and to use alternative ways as shown in Fig. 1**d** under the facilitator teaching style. For *S1, facts, concepts, and principles are the most important things that students should acquire,* 115 (46.37%) teachers moderately agreed with this teaching style as shown in Fig. 1**a** under expert teaching style. For S40, *I assume the role of a resource person who is available to students whenever they need help,* 108 (43.54) teachers moderately disagreed and 120 (48.38) strongly disagreed, respectively as shown in Fig. 1**e** under delegator teaching style.

The results of the Chi-square test showed that all statements were significant reflecting that the observed frequencies of teacher’s responses are statistically significant from expected frequencies within each category (*X*^*2*^ (4, *N* = 248) = 29.09 ~ 549.61, *p* < 0.0001) as shown in **Supplementary Table** **1**.

Table 1 shows distribution and reability of different groups of teaching styles as calculated by cronbach alpha in our study. The tables also compares reliability of different clusters of teaching styles in our study with two other published reports [19] [20]. These results reflect that reliability of each teaching is higher than the cutpoint of 0.70 and similar comparable results were found in other two studies as well. Table 2 presents summary statistics of teaching styles, demographic and academic variables recorded in our study. Among the teaching styles, delegator teaching style was the most common variant as shown by high median and mean of 2.38 and 2.45, respectively.

**Table 1:** Distribution and reliability of different teaching styles as calculated by Cronbach’s alpha in our study as compared with other two published reports

Teaching styles	Items	Reliability	Reliability [19]	Reliability [20]
**Expert**	1–6–11-16-21-26-31-36	**0.73**	0.78	0.73
**Authority**	2–7–12-17-22-27-32-37	**0.82**	0.82	0.80
**Demonstrator**	3–8–13-18-23-28-33-38	**0.76**	0.74	0.71
**Facilitator**	4–9–14-19-24-29-34-39	**0.78**	0.80	0.75
**Delegator**	5–10–15-20-25-30-35-40	**0.82**	0.72	0.83

**Table 2:** Descriptive characteristics of the study groups

Characteristics	***N*** (%)	Mean	Median	Std. deviation	Minimum	Maximum
**Gender**						
Female	114 (46%)					
Male	134 (54%)					
**Course level**						
Higher	94 (38%)					
Lower	154 (62%)					
**Academic rank**						
Senior	190 (77%)					
Junior	58 (23%)					
**Teaching experience**						
> 5 years	134 (54%)					
< 5 years	114 (46%)					
**Teaching institute**						
University of Sharjah	25 (10%)					
University Sains Malaysia	5 (6%)					
Imam Abdulrahman Bin Faisal University	67 (27%)					
Maastricht University	47 (19%)					
Taibah University	35 (14%)					
University of Gezira	49 (20%)					
Shifa Tameer-e-Millat University	10 (4%)					
**Teaching style**						
Expert	248	2.27	2.25	0.66	1	4.62
Authority	248	1.98	1.94	0.59	1	4.88
Delegator	248	2.45	2.38	0.56	1.25	5
Demonstrator	248	1.83	1.75	0.6	1	4.75
Facilitator	248	2.15	2.13	0.59	1	4.88

Note: Variables definitions are as follows: teachings styles are continuous variables measured by taking the average of statements for each style as shown in Table 1. Gender was taken as 1 for women and 0 for men. Course level was considered as 1 for respondents who taught senior students (years 4 and 5) and 0 for teachers of junior students (years 1, 2 and 3). Academic rank was taken as 1 for senior teachers (professor, associate, and assistant professor) and 0 for junior teachers (lecturer, adjunct faculty). Teaching experience was taken as 1 for senior teachers with more than 5 years’ experience and 0 for juniors with less than 5 years’ experience. Finally the variable UoS was considered as 1 if responding teacher represented University of Sharjah and 0 otherwise; likewise, other institutions were given 1 and 0 using the same coding system

The results of the Pearson’s correlation analysis of different teaching styles, demographics and academic variables are shown in Table 3. Interestingly, we found a signifcant correlation between different teaching styles, among which facilitator and demonstrator teaching styles have highest correlation coefficient value of 0.75 which is significant at 1% level of significance. Regarding the correlation between teaching styles and demographics, we found a significant coefficient value of − 0.17 between authority teaching style and gender, which indicates that females were less authoritative as compared to their male counterparts. We have also found a signifncant relationship obetween academic rank with expert and delegate teaching styles. Table 4 demonstrates the results of multiple regression analysis of different teaching styles, demographics and academic variables. Among the demographic variables and teaching styles, our computed results showed that only authority teaching style and gender had significantly relationship (coefficient value − 0.20), which indicates that females were 20% less authorative in teaching compared to male teachers. In addition, results showed a signifncant relationship between academic rank and expert and delegate teaching styles with coefficent values of 0.217 and 0.175, respectively. This reflects that seniors are 21.7 and 17.5% more experts and delegate teachers as compared to junior teachers. Finally, reagaring medical schools, we found signifncant correlation among teachers from STMU as compared to other medical schools.

**Table 3:** Correlation analysis using the Pearson’s correlation test for different teaching styles, demographics and academics (N = 248)

Features	1	2	3	4	5	6	7	8	9	10	11	12	13	14	15	16
1. Expert	–															
2. Authority	0.68^**^	–														
3. Delegator	0.63^**^	0.63^**^	–													
4. Demonstrator	0.46^**^	0.72^**^	0.43^**^	–												
5. Facilitator	0.46^**^	0.61^**^	0.47^**^	0.75^**^	–											
6. Gender	−0.10	−0.17^**^	−0.08	−0.04	0.01	–										
7. Course level	−0.04	− 0.05	− 0.07	− 0.10	− 0.11	− 0.21^**^	–									
8. Academic rank	0.12**	0.02	0.08**	−0.04	− 0.03	− 0.26^**^	− 0.17^**^	–								
9. Teaching experience	0.02	0.06	−0.01	0.03	0.02	−0.05	0.08	.294^**^	–							
10. UoS	−0.06	0.05	0.04	0.08	0.10	−0.05	−0.14^*^	− 0.01	−0.07	–						
11. USM	0.03	0.03	−0.03	0.09	0.09	0.11	0.01	−0.17^**^	0.03	−0.09	–					
12. IAU	0.01	0.01	0.08	0.02	0.01	0.13	0.01	−0.11	− 0.08	− 0.20^**^	− 0.16^*^	–				
13. MU	−0.08	−0.13^*^	− 0.14^*^	− 0.10	− 0.10	0.03	0.09	−0.09	0.11	−0.16^*^	− 0.13	− 0.29^**^	–			
14. TU	−0.02	−0.08	− 0.09	−0.04	− 0.03	0.08	− 0.29^**^	0.23^**^	0.25^**^	−0.13^*^	− 0.11	− 0.24^**^	− 0.19^**^	–		
15. UoG	0.01	0.01	−0.04	−0.13	− 0.13^*^	−0.27^**^	0.31^**^	0.17^**^	−0.15^*^	−0.16^*^	− 0.13	−0.29^**^	− 0.23^**^	−0.19^**^	–	
16. STMU	0.15^*^	0.20^**^	0.24^**^	0.16^*^	0.14^*^	−0.02	− 0.13^*^	− 0.11	− 0.12	−0.07	− 0.06	−0.13^*^	− 0.11	−0.09	− 0.11	–

Note: ** and * represent 1 and 5% level of significance

Note: Variables definitions are as follows: *teachings styles* are continuous variables measured by taking the average of statements for each style as shown in Table 1. *Gender* was taken as 1 for women and 0 for men. *Course level* was considered as 1 for respondents who taught senior students (years 4 and 5^)^ and 0 for teachers of junior students (years 1,2,3). *Academic tank* was taken as 1 for senior teachers (professor, associate, assistant professor) and 0 for junior teachers (lecturer, adjunct faculty). *Teaching experience* was taken as 1 for senior teachers with more than 5 years’ experience and 0 for juniors with less than 5 years’ experience. Finally the variable *UoS* was considered as 1 if responding teacher represented UoS and 0 otherwise; Likewise, other institutions were given 1 and 0 using the same coding system

*UoS* University of Sharjah, *USM* University Sains Malaysia, *IAU* Imam Abdulrahman Bin Faisal University, *MU* Maastricht University, *TU* Taibah University; University of Gezira, *STMU* Shifa Tameer-e-Millat University

**Table 4:** Multiple regression analysis of different teaching styles, demographics and academics (*N* = 248)

Features	(1)	(2)	(3)	(4)	(5)
	Expert	Authority	Delegator	Demonstrator	Facilitator
Gender	−0.117	− 0.200**	− 0.069	−0.081	− 0.021
	(0.092)	(0.077)	(0.074)	(0.079)	(0.080)
Course level	−0.135	− 0.116	−0.118	− 0.107	−0.114
	(0.100)	(0.084)	(0.082)	(0.086)	(0.087)
Academic rank	0.217***	0.003	0.175**	−0.019	0.020
	(0.103)	(0.095)	(0.081)	(0.097)	(0.098)
Teaching experience	0.014	0.129	0.017	0.058	0.048
	(0.094)	(0.079)	(0.076)	(0.081)	(0.082)
USM	0.338	0.046	−0.016	0.094	0.039
	(0.218)	(0.184)	(0.176)	(0.188)	(0.190)
IAU	0.209	−0.026	0.069	−0.087	− 0.141
	(0.159)	(0.134)	(0.128)	(0.137)	(0.138)
MU	0.092	−0.221	−0.158	− 0.234	−0.269*
	(0.170)	(0.143)	(0.137)	(0.146)	(0.147)
TU	0.048	−0.239	−0.222	−0.221	− 0.257
	(0.182)	(0.154)	(0.147)	(0.157)	(0.158)
UoG	0.158	−0.050	−0.078	− 0.244	−0.283*
	(0.173)	(0.146)	(0.140)	(0.149)	(0.150)
STMU	0.605**	0.422**	0.512***	0.291	0.154
	(0.236)	(0.199)	(0.190)	(0.203)	(0.205)
Constant	2.043***	2.097***	2.407***	2.002***	2.308***
	(0.164)	(0.139)	(0.133)	(0.142)	(0.143)
Observations	248	248	248	248	248
R-squared	0.165	0.207	0.206	0.178	0.066

Note: Standard errors in parentheses, *** *p* < 0.01, ** *p* < 0.05, * *p* < 0.1

Note: Variables definitions are as follows: *teachings styles* are continuous variables measured by taking the average of statements for each style as shown in Table 1. *Gender* was taken as 1 for women and 0 for men. *Course level* was considered as 1 for respondents who taught senior students (years 4 and 5^)^ and 0 for teachers of junior students (years 1,2,3). *Academic tank* was taken as 1 for senior teachers (professor, associate, assistant professor) and 0 for junior teachers (lecturer, adjunct faculty). *Teaching experience* was taken as 1 for senior teachers with more than 5 years’ experience and 0 for juniors with less than 5 years’ experience. Finally the variable *UoS* was considered as 1 if responding teacher represented UoS and 0 otherwise; Likewise, other institutions were given 1 and 0 using the same coding system

*UoS* University of Sharjah, *USM* University Sains Malaysia, *IAU* Imam Abdulrahman Bin Faisal University, *MU* Maastricht University, *TU* Taibah University; University of Gezira, *STMU* Shifa Tameer-e-Millat University

## Discussion

In this international multicenter study, though, we observed agreements about majority of the suggested teaching styles; however, significant variations were reported across level of experience of faculty, gender and institutions. This study reports the delegator teaching style as the most popular variant, a strong correlation between expert and authority teaching styles, and a significant correlation between male gander and authority teaching style. Within the five classic teaching styles of Grasha, the expert, authority and demonstrator styles match with traditional and teacher-centered teaching approach, while facilitator and delegator styles fall under facilitative and student-centered approach [21]. Additionally, in higher education, mostly teachers follow a mulit-modal teaching approach. Therefore, each teacher possesses some elments from each of the five teaching styles to varying degrees.

Compared to the delegator teaching style as the most preferred approach, Razak et al. have identified a multi-modal pattern of expert, demonstrator and delegator styles [22]. This findings by Razak et al. are congruent with Grasha’s results that higher education teachers tend to prefer a blend a teaching styles [23]. Nevertheless, one dominat teaching style would be easily reflected in their practice. In the study by Harden and Crosby, the authors have emphasized on six key roles of a good medical teacher; information provider, role model, facilitator, student assessor, curriculum planner and resource material provided [24]. Of these facilitator role of a medical teacher matches with the delegator styles as both approaches empower students to set the pace and direction of their learning. Among the academic variables and teaching styles, our study showed significant correlation between expert and authority teaching styles and nature of curriculumn (coefficent value of 0.145 and 0.30, respectively). This is an interesting observation. All particiapnts in our study belonged to medical schools with PBL curriculum. As PBL tutor, the leading role of medical teachers is based on facilitation of students’ learning process rather than to deliver knowledge [25]. However, due to the structural hetrogenity of PBL curriculum, the role of teacher shifts from a facilitator to information provider to an evaluator [26] . This partly explains the finding of a bimodal pattern of expert and authority teaching styles in our study.

Among several unique observations, the commonest teaching approach with 195 (68%) agreement was reported for the strategy where students’ learning was facilitated by allowing them to raise questions and to use alternative ways of group discussions and peer learning (S19). The participants also agreed about facilitative roles of medical teachers in soliciting students, small group learning, role modeling and in providing effective feedback (S14,S15). Small group teaching promotes peer learning for acquisition of knowledge and skills [27], while role modeling carries valuable impact on the professional and character development and career evolution of the modelees [28]. In addition, role modeling is a vital element in nurturing desired characteristics of professionalism in a hidden curriculum [29]. The consensus of majority of medical teachers on such educational strategies can be considered as the basis for a positional framework of recommended teaching styles as an end-point outcome from our study.

Analyzing the most preferred and practiced teaching style from each university, we have identified different teaching styles that were distinct to each institution. Of the Arabian universities, the faculty in UoS, UAE preferred to provide students with more freedom in content, design and self-directed learning (S15). Faculty in TU Saudi Arabia provided a broader perspective of topics to the students (S11), while faculty in UoG, Sudan were more motivated to set standards in class and encourage their students to develop their own ideas (S2, S8). The faculty in IAU Saudi Arabia encouraged their students to develop their own learning profile (S8). Among the non-Arabian universities, faculty in USM, Malaysia were more inclined to spend time with their students both at individual and group levels to improve their learning and work experiences (S9). On the other hand, faculty in MU, the Netherlands preferred small group discussions to develop student’s ability to think critically (S14). Lastly, faculty STMU, Pakistan would help the students to resolve disagreement about content issues and student perceived faculty more as a “coach” than a teacher (S38). The core theme of preferred teaching styles from all institutions endorse a student-centered education with more time and efforts dedicated to individual mentoring. The variations in teaching styles most likely signify diverse cultural and regional issues differences. In their quantitative and qualitative evaluations of teachers’ effectiveness by students, Kassab et al. have reported that tutors recognize themselves as facilitators and collaborators, while students considered tutors as assertive personnel. The authors have deduced that there was a disparity between students’ and tutors’ opinions about tutor’s teaching styles. Teachers’ experience of effective teaching by the faculty when they were students and responses to feedback from students are other important predictors of effective teaching preferrnecs.

Interestingly, majority of respondents in our study agreed with the range of teaching styles outlined in the questionnaire. However, UoG senior faculty showed strongest agreement with the teaching style where teacher’s goals and methods articulate with a variety of students learning styles (*p* = 0.000). In medicine, physician educators attain their teaching skills without any formal training. In our study, the responses of adjunct faculty attached significant value to lecturing as their core teaching style. This observation highlights the value of faculty development programs in providing structured training and teaching skills [30]. These programs help the medical educators in their transition from teacher to a facilitator, curriculum planner, evaluator, researchers and scholar, multitasking professionals [31]. A gradual deviation from classical apprenticeship model of teaching towards a more student-centered teaching legacy in an inter professional climate has also been advocated in medical education [32]. This premise is quite evident in multi-center study, though faculty from TU and STMU were less inclined towards empowering students to set pace of their learning process.

Our study has shown differences in preferered teaching styles between more experienced (> 10 years) and less experienced teachers (< 10 years)**.** Young teachers were able to align their teaching methods closely to a variety of student’s learning styles. The young teachers also believed in role modelling, use of appropriate technologies to match student’s learning styles, and did not hesitate to provide negative feedback for unsatisfactory performance. Awareness about medical students’ learning styles is a powerful teaching strategy that can help educators in customizing instructional methods for enhancing students’ knowledge and competence [33]. Knowledge of learning preferences of students supports educators in developing teaching and learning strategies for conducive classroom environment. Conversely, Paiboonsithiwong et al. have argued that matching students’ learning styles with teaching styles are not associated with better academic achievement or in alleviating mental health [34]. Regarding role modelling, Passi et al. have reported that personality characteristics of positive role models influence students’ professional development and career choice [35]. Nevertheless, culture, diversity, experience and gender pose major impact on choice of role model. Our study reflected that male teachers in younger age groups rely on achievement of specific goals and objectives of their taught course. Teaching experience has been shown to be positively correlated with effective teaching [36].

We observed significant variations in teaching style preferences among male and female teachers. Soliciting students, aligning teaching with student learning styles and setting standards for students’ learning were perceived differently by both genders. While male teachers allowed students more autonomy by reducing direct supervision and setting their own pace of doing course work, female teachers were more willing to ask their students about content and process of teaching. Although differences in preferred teaching styles between male and female health professionals have not been well researched, however, reports from primary education signal clear differences in teaching styles [37]. Male teachers encourage teamwork, prefer non-verbal communication and provide freedom to the learners [38]; whereas female teachers prefer interpersonal and verbal communication and apply pedagogical dialogue in achieving educational goals [39, 40].

Our study about teaching styles of medical teachers includes data of medical faculties with wide range of experience, different genders, and academic ranks. Heterogeneity of this data might not enable the researchers to draw generalizable conclusions for harmonizing preferred teaching styles due to diverse community needs and versatile faculty expertise. Further studies are required to focus on explicit dimensions of teaching styles that will help in developing targeted faculty training and enhancement programs.

## Conclusion

This international multicenter study is first of its kind that has compared preferred teaching styles of medical faculty from seven institutions in six countries. Most participants preferred delegator teaching style. We report congruence on teaching styles that embrace small group teaching, self-directed learning, role modelling, and facilitative role of teachers. Cultural, educational, gender, experience and types of medical curricula might influence some of the indentified diverging teaching styles such as strictly following course contents. Medical educators can use this valuable data to calibrate their teaching styles for accommodating diverse needs of medical students.

## Supplementary Information

**Additional file 1. **Supplementary Table 1. Results of chi-square test of independence for different teaching styles (*N* = 248)

## Data Availability

Not applicable.

## Abbreviations

UAE: United Arab Emirates
UOS: University of Sharjah
UOG: University of Gezira
USM: Universiti Sains Malaysia
MU: Maastricht University, the Netherlands
TU: Taibah University
IAU: Imam Abdulrahman Bin Faisal University
STMU: Shifa Tameer-e-Millat University
