# Supplementary material for: Preferred teaching styles of medical faculty: an international multi-center study
Source: BMC Med Educ. 2020 Nov 30;20:480. doi: 10.1186/s12909-020-02358-0 (PMC7708200; doi:10.1186/s12909-020-02358-0)
Supplement: Supplementary file 1 — Additional file 1. Supplementary Table 1. Results of chi-square test of independence for different teaching styles (N = 248) [file 12909_2020_2358_MOESM1_ESM.docx]

Supplementary Table 1. Results of chi-square test of independence for different teaching styles (N=248)

|  | Chi-Square | df | Asymp. Sig. |
| --- | --- | --- | --- |
| 1. Facts, concepts, and principles are the most important things that students should acquire. | 174.984^a^ | 4 | 0.000 |
| 2. I set standards for students in my class. | 176.122^b^ | 4 | 0.000 |
| 3. What I say and do models appropriate ways for students to think about facts and principles in the content. | 220.347^a^ | 4 | 0.000 |
| 4. My teaching goals and methods are compatible with a variety of students learning styles. | 60.297^c^ | 4 | 0.000 |
| 5. Students typically work on course projects alone with little supervision from me. | 412.740^d^ | 4 | 0.000 |
| 6. Sharing my knowledge and expertise with students is very important to me. | 64.835^c^ | 4 | 0.000 |
| 7. I give students negative feedback when their performance is unsatisfactory. | 230.739^c^ | 4 | 0.000 |
| 8. Activities in my class encourage students to develop their own ideas about content issues. | 206.840^e^ | 4 | 0.000 |
| 9. I spend time consulting with students on how to improve their work on individual and/or group projects. | 232.720^e^ | 4 | 0.000 |
| 10. Activities in my class encourages students to develop their own ideas about content issues. | 244.840^e^ | 4 | 0.000 |
| 11. What I have to say about a topic is important for students to acquire a broader perspective on the issues in that area. | 43.831^c^ | 4 | 0.000 |
| 12. In my opinion, students would describe my standards and expectations as somewhat strict and rigid. | 135.800^e^ | 4 | 0.000 |
| 13. I typically show students how and what to do in order to master course content. | 376.964^c^ | 4 | 0.000 |
| 14. Small group discussions are employed to help students develop their ability to think critically. | 89.800^e^ | 4 | 0.000 |
| 15. Students design one or more self-directed sessions. | 439.280^e^ | 4 | 0.000 |
| 16. I want students to leave this course well prepared for further work in this area. | 76.522^c^ | 4 | 0.000 |
| 17. It is my responsibility to define what students must learn and how they should learn it. | 248.600^e^ | 4 | 0.000 |
| 18. I often use examples from my personal experiences to illustrate points about the material. | 306.680^e^ | 4 | 0.000 |
| 19. I guide students' work on course projects by asking questions, exploring options, and suggesting alternative ways to do things. | 549.614^c^ | 4 | 0.000 |
| 20. Developing the ability of students to think and work independently is an important goal. | 59.661^a^ | 4 | 0.000 |
| 21. Lecturing is a significant part of how I teach each of the class sessions. | 130.260^d^ | 4 | 0.000 |
| 22. I provide very clear guidelines for how I want tasks completed in this course. | 216.234^a^ | 4 | 0.000 |
| 23. I often show students how they can use various principles and concepts. | 325.357^c^ | 4 | 0.000 |
| 24. Course activities encourage students to take initiative and responsibility for their learning. | 170.710^a^ | 4 | 0.000 |
| 25. In my opinion, students take responsibility for teaching part of the class sessions. | 117.673^b^ | 4 | 0.000 |
| 26. My expertise is typically used to resolve disagreements about content issues. | 249.702^a^ | 4 | 0.000 |
| 27. This course has very specific goals and objectives that I want to achieve. | 162.737^f^ | 4 | 0.000 |
| 28. Students receive frequent verbal and/or written comments on their performance. | 92.211^f^ | 4 | 0.000 |
| 29. I solicit student advice about how and what to teach in this course. | 103.024^d^ | 4 | 0.000 |
| 30. Students set their own pace for completing independent and/or group projects. | 48.129^c^ | 4 | 0.000 |
| 31. Students might describe me as a "storehouse of knowledge" who dispenses the fact, principles, and concepts they need. | 182.107^g^ | 4 | 0.000 |
| 32. My expectations for what I want students to do in this class are clearly defined in the syllabus. | 113.992^c^ | 4 | 0.000 |
| 33. Eventually, many students begin to think like me about course content. | 108.851^c^ | 4 | 0.000 |
| 34. Students can make choices among activities in order to complete course requirements. | 29.093^f^ | 4 | 0.000 |
| 35. My approach to teaching is similar to a manager of a work group who delegates tasks and responsibilities to subordinates. | 44.016^a^ | 4 | 0.000 |
| 36. There is more material in this course than I have time available to cover it. | 149.944^a^ | 4 | 0.000 |
| 37. My standards and expectations help students develop the discipline the need to learn. | 134.782^a^ | 4 | 0.000 |
| 38. Students might describe me as a "coach" who works closely with someone to correct problems in how they think and behave. | 322.615^f^ | 4 | 0.000 |
| 39. I give students a lot of personal support and encouragement to do well in this course. | 232.120^e^ | 4 | 0.000 |
| 40. I assume the role of a resource person who is available to students whenever they need help. | 171.184^h^ | 3 | 0.000 |

**Note:** The minimum expected cell frequency: a = 49.6; b = 49.0; c = 49.8; d = 49.2; e = 50.0; f = 49.4; g = 48.8; and h = 62.5. Total respondents (n = 248) and * represents p value < 0.01.
